# Retrospective cohort study assessing cardiovascular risk reduction through intensive blood pressure management in high-risk patients

**DOI:** 10.3389/fcvm.2026.1711504

**Published:** 2026-06-17

**Authors:** Baihui Tang, Wenxi Gu

**Affiliations:** Department of Cardiology, Affiliated Chongming Hospital of Shanghai University of Medicine & Health Sciences, Shanghai, China

**Keywords:** cardiovascular risk reduction, chronic kidney disease, diabetes, high-risk patients, hypertension, intensive blood pressure management, retrospective cohort study

## Abstract

**Background:**

Hypertension remains a major global risk factor for cardiovascular disease. While clinical trials suggest that lower systolic blood pressure (SBP) levels reduce cardiovascular risk, real-world data from high-risk patients remain limited. This study evaluated the association between achieved SBP levels and cardiovascular outcomes in a high-risk hypertensive population.

**Methods:**

We performed a retrospective cohort study from January 2022 to December 2024, including 180 high-risk hypertensive patients receiving treatment at a tertiary care facility. Patients were categorized based on achieved systolic blood pressure during follow-up: one group with SBP <120 mmHg and another with SBP between 130 and 139 mmHg. The primary endpoint was a composite of major adverse cardiovascular events (MACE), including myocardial infarction, stroke, heart failure, and cardiovascular-related death or hospitalization. The secondary outcomes consisted of all-cause mortality and adverse treatment events.

**Results:**

Patients in the lower achieved SBP group had significantly fewer MACE events over a median follow-up of 18 months compared with those in the higher SBP group (14.8 vs. 26.5, *p* = 0.03). Treatment was considered to be particularly of benefit for patients with diabetes and chronic kidney disease because the relative risk reduction was 30% compared to routine control. There was attenuation of benefits in elderly patients, i.e., where age was over 75. Adverse events, including hypotension and renal impairment, were slightly more common in the lower SBP group but remained clinically manageable (9.3% vs. 4.8%; *p* = 0.60).

**Conclusion:**

Among high-risk hypertensive patients, lower achieved SBP levels were associated with reduced cardiovascular event rates. These findings support the need for further prospective studies to define optimal BP targets, especially in patients with diabetes and chronic kidney disease.

## 1 Introduction

Hypertension is a global public health concern. It is one of the most important risk factors of cardiovascular disease (1). GBD 2021 reported that more than 10 million people die every year because of hypertension- more than 1.3 billion people worldwide live with the disorder, the major contributors being ischemic heart disease, stroke, and heart failure (2). The situation is worsening year on year due to further aging of the population, the expansion of urban populations, the adoption of sedentary lifestyles, and high-risk dietary behavior (high sodium and low potassium intake). The number of people suffering from hypertension is expected to reach 1.5 billion by the year 2030 (3), which has reached an alarming level and has serious consequences on the economy and the health care system.

Hypertension is a problem that has an even more detrimental effect on population health in high-risk groups such as people with diabetes, chronic kidney disease (CKD), and people with established atherosclerotic cardiovascular disease (4, 5). In these populations, the public health consequences of hypertension are immeasurable. They are accompanied by an increase of reno-vascular and aortic systolic and mean arterial pressures, and as such are accompanied by far more rapid left ventricular hypertrophy, impaired central nervous system, and other vital organ hemodynamics, significantly increasing the risk of stroke, renal failure, myocardial infarction, and untimely death (6–8). The association of kidney and metabolic syndrome, and hypertension is the most serious and commonplace, and the one that most often advances cardiovascular disease (9). The residual risk of cardiovascular disease for poorly managed hypertension further justifies the necessity for optimizing treatment plans (10). While all patients stand to benefit, high-risk individuals need specific management that optimally balances benefit and risk. This gap in evidence has also driven the need to refocus on the better management of hypertension, that is, holding more aggressive treatment of hypertension to lower systolic blood pressure to levels lower than the thresholds set in usual care to lower cardiovascular disease morbidity and mortality more effectively.

In the last twenty years, the approach to managing hypertension has changed drastically from non-invasive to risk-based. Most guidelines from JNC 7 suggested less than 140 mmHg as the SBP target for the majority of the population and less than 130 mmHg for people with diabetes or chronic kidney disease (11). But then the risk of cardiovascular event became associated with ‘residual’ risk, and the question became whether we are doing enough to mitigate cardiovascular risk with these targets. A turning point was the Systolic Blood Pressure Intervention Trial (SPRINT, 2015), which showed that having a target of less than 120 mmHg SBP significantly reduced most cardiovascular outcomes and all-cause mortality as compared to the less than 140 mmHg target (12). Targeting these levels of SBP became especially important in people with heightened cardiovascular risk, in the absence of diabetes and previous stroke (13). Global thinking and other professional societies shifted significantly after these findings, and some of them started to change their guidelines. For instance, the 2017 guidelines from the American College of Cardiology/American Heart Association (ACC/AHA) started to lower the threshold for hypertension diagnosis to 130/80 mmHg and suggested eliminating the more conservative targets in these populations (14). All things considered, the advancement of blood pressure control has changed from a one-size-fits-all approach to setting individualized treatment goals that take into account the patient's level of risk. Although the evidence is overwhelmingly supportive of the cardiovascular benefits of intensive therapy in many high-risk groups, its use in older or frail patients requires careful consideration of the risks and benefits (15). These intricacies have provided the justification for continuous research, including retrospective and real-world studies like the current one, so that optimal treatment modalities for various patient populations can be developed.

Despite the promising results, the literature still has significant holes. For starters, most randomized controlled trials were done in confined environments, making the results less applicable to ‘real’ patients, particularly in the case of those with multiple complex conditions and frailty. The debate about the impact of intensive blood pressure lowering in the elderly still lingers, with some studies showing less favorable or even more harmful results in this population. In addition, there is very little retrospective information available to assess the impact of intensive blood pressure management on the day-to-day practice of clinical medicine, particularly in settings where there is low adherence, high comorbidity, and polypharmacy.

Considering these uncertainties, retrospective approaches can shed light on the question of the effectiveness and safety of intensive blood pressure control in certain at-risk populations. In light of more recent evidence, we attempted to assess the relationship between BP control and cardiovascular outcomes in a real-world setting among intensive BP-managed, high-risk hypertensive patients from 2022 to 2024. We assumed that intensive management would lower the cardiovascular risk more than standard care merited while keeping the safety within a reasonable limit in order to sustain the practice of treatment target optimization.

## 2 Materials and methods

### 2.1 Study design and setting

This was a retrospective cohort study conducted between January 2022 and December 2024 at Affiliated Chongming Hospital of Shanghai University of Medicine & Health Sciences, Shanghai, China. The study aimed to evaluate the association between achieved systolic blood pressure levels and cardiovascular outcomes in high-risk hypertensive patients. Patient data were extracted from institutional medical records and electronic health databases.

Patients were not assigned to treatment arms prospectively. Instead, they were retrospectively categorized based on the average systolic blood pressure achieved during follow-up. This grouping reflected real-world treatment responses rather than predefined BP targets.

### 2.2 Study population

**Figure 1:**
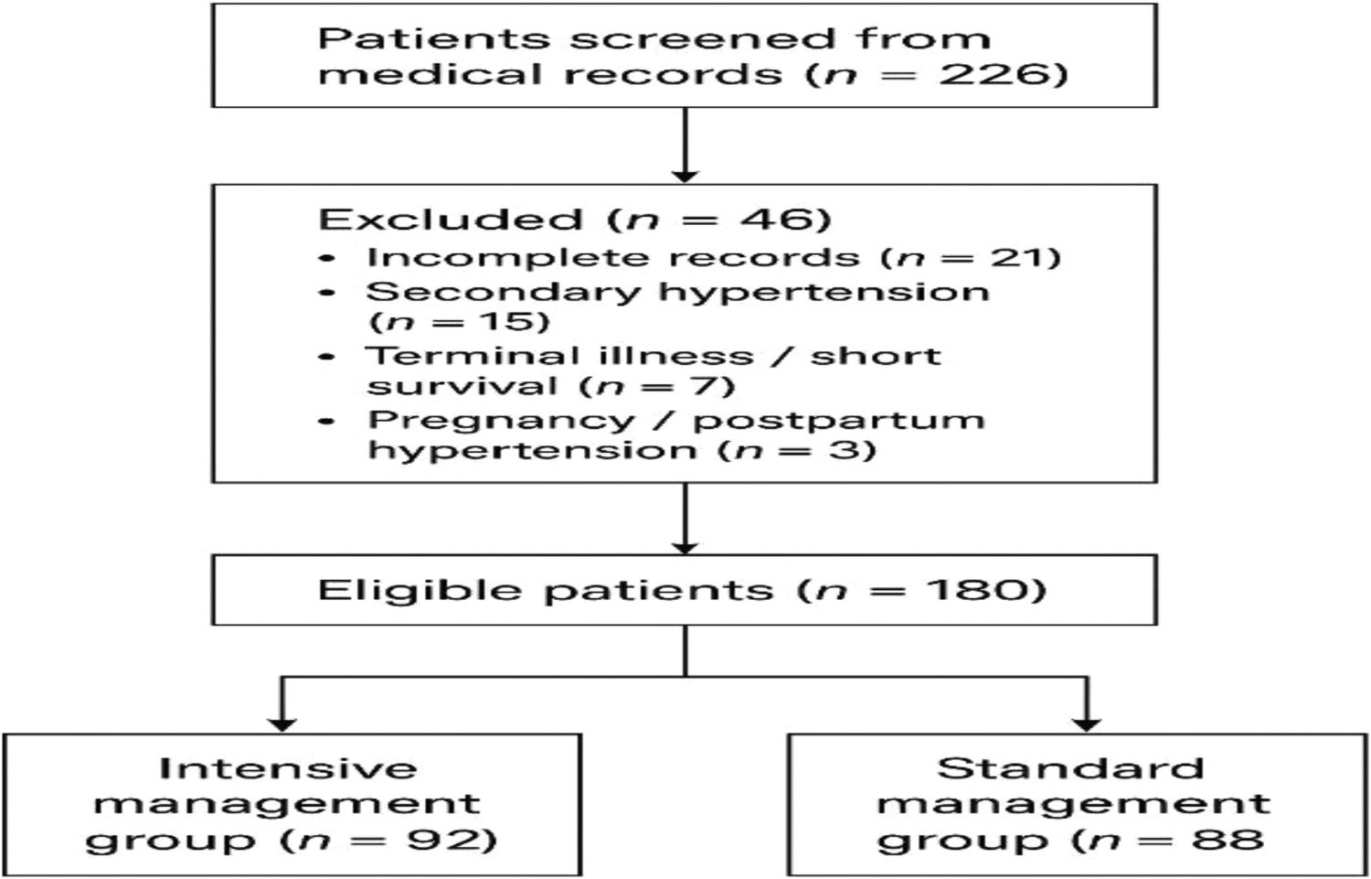
Study flow diagram.

The study group included around 180 patients at high risk of health issues who were eligible for the study from January 2022 to December 2024 (Figure 1). Among them, only people who were 40 and above and were diagnosed with hypertension with at least one other risk of cardiovascular disease, like diabetes, chronic kidney disease, ischemic heart disease, or were active smokers, were considered. Only patients with two or more visits to the clinic for the entire duration of the study and with the required documentation, both baseline and follow-up, were placed into analysis. Exclusions were made if there was missing clinical data, if hypertension was due to endocrine or renovascular issues, and if there were other conditions that would mean the patient would only live for the next 6 months. Women who were pregnant and diagnosed with postpartum hypertension were also removed due to potential outcome confounding.

### 2.3 Data collection and variables

The data retrieval process was performed on the electronic health records of the patient and cross-referenced with the identified medical records for accuracy to ensure accuracy. Over the years, patients have collected data such as age, gender, body mass index, diabetes, and other information, which was later supplemented with other clinical parameters, including hiatus and other medical histories. Patients were divided into two treatment groups: those with diabetes and those with managed diabetes. Those who managed diabetes were further divided into two groups: intense control and standard control, with the former focusing on the systolic blood pressure. Follow-up data determined that on average, patients reported a minimum of 12 months of cardiovascular events such as heart attacks, strokes, heart failure, and cardiovascular death. Further record reviews about kidney and heart status, blood lipids, and sugar levels were reviewed to assess the later consequences coming from diabetes and other management intensively.

### 2.4 Blood pressure categories and outcome measures

Patients were retrospectively categorized into two groups based on their achieved systolic blood pressure (SBP) during the follow-up period, as documented in their medical records. The intensive group consisted of patients whose treatment focused on achieving an SBP of 120 mmHg. The standard management group consisted of patients who were capped at a systolic pressure of 140 mmHg. It is important to note that this grouping was not the result of any predefined treatment assignment or protocol-driven target; rather, it reflects real-world variations in treatment response and clinical management practices. The main outcome of interest was the occurrence of major adverse cardiovascular events (MACE), which was a combination of having a heart attack, stroke, being admitted with heart failure, and dying from heart disease. Secondary outcomes included individual cardiovascular endpoints, changes in kidney health, and symptoms and complications of treatment, like hypotension, electrolyte imbalance, or kidney failure. Outcomes were retrieved from medical documents, discharge summaries, laboratory results, and electronic health records.

### 2.5 Statistical analysis

All analyses were performed using SPSS version. Continuous variables were expressed as mean ± standard deviation and compared using the independent-samples t-test, while categorical variables were presented as frequencies (%) and analyzed using the chi-square test. Kaplan assessed event-free survival–Meier curves with the log-rank test. Multivariable Cox proportional hazards regression was used to adjust for baseline confounders. A *p*-value < 0.05 was considered statistically significant.

## 3 Results

### 3.1 Anti-proliferative activity of vanoxerine in AML cells

All 180 participants in the final analysis were retrospectively categorized into 2 groups based on their achieved systolic blood pressure (SBP) during the follow-up period. The lower SBP group (*n* = 92) consisted of patients with an average SBP <120 mmHg, and the higher SBP group (*n* = 88) included patients with SBP between 130 and 139 mmHg. These categories reflect observed blood pressure outcomes and do not indicate prospective treatment allocation. The overall mean age in the study population was 62.4, and there were no major age differences between the 2 groups. Approximately 58% of the study participants were male. The presence of diabetes was higher than average with 46% of patients, chronic kidney disease was 28%, and 32% had a history of ischemic heart disease. The average systolic blood pressure in the standard management group was higher (mean 151.2 ± 12.4 mmHg) than the intensive management group (mean 148.6 ± 11.7 mmHg), but this difference was not clinically meaningful (*p* = 0.08). Both the body mass index and renal function, along with lipid and blood pressure profiles, were fairly matched between the two groups, suggesting adequate balance in the participants’ individual attributes at the time of enrollment (Table 1).

**Table 1:** Baseline characteristics of the study population.

Characteristic	Lower SBP group (*n* = 92)	Higher SBP group (*n* = 88)	*p*-value
Age, years (mean ± SD)	62.1 ± 9.5	62.8 ± 10.1	0.64
Male sex, *n* (%)	54 (58.7)	50 (56.8)	0.78
Body mass index, kg/m^2^ (mean ± SD)	27.4 ± 3.2	27.9 ± 3.4	0.41
Diabetes mellitus, *n* (%)	43 (46.7)	40 (45.5)	0.88
Chronic kidney disease, *n* (%)	25 (27.2)	26 (29.5)	0.72
Ischemic heart disease, *n* (%)	31 (33.7)	27 (30.7)	0.68
Current smoker, *n* (%)	19 (20.7)	18 (20.5)	0.97
Baseline SBP, mmHg (mean ± SD)	148.6 ± 11.7	151.2 ± 12.4	0.08
Baseline DBP, mmHg (mean ± SD)	86.3 ± 8.5	87.1 ± 8.8	0.54
Serum creatinine, mg/dL (mean ± SD)	1.19 ± 0.32	1.22 ± 0.34	0.61
LDL cholesterol, mg/dL (mean ± SD)	118.4 ± 29.7	120.1 ± 30.2	0.74

In the follow-up period, which, on average, was 18 months long (IQR: 14–22 months), those patients in the ‘intensive management’ cohort were able to achieve substantially lower mean systolic blood pressure levels relative to those in the ’standard management’ cohort (122.8 ± 6.4 mmHg vs. 136.5 ± 7.2 mmHg, *p* < 0.001). In the intensive group, the proportion of patients achieving the assigned target was greater (88%) than in the standard group (74%) (Table 2). Furthermore, the intensive management group demonstrated a greater reduction in diastolic blood pressure, although the difference was not nearly as pronounced as the reduction of systolic blood pressure.

**Table 2:** Achieved blood pressure and clinical outcomes during follow-up in lower vs. Higher SBP groups.

Variable	Lower SBP group (*n* = 92)	Higher SBP group (*n* = 88)	*p*-value
Mean follow-up duration, months (median, IQR)	18 (14–22)	18 (14–21)	0.79
Mean SBP at follow-up, mmHg (± SD)	122.8 ± 6.4	136.5 ± 7.2	<0.001
Mean DBP at follow-up, mmHg (± SD)	77.2 ± 5.6	81.1 ± 6.1	0.002
Achieved target BP, *n* (%)	81 (88.0)	65 (73.9)	0.01
Reduction in SBP from baseline, mmHg	−25.8 ± 8.6	−14.7 ± 7.9	<0.001
Reduction in DBP from baseline, mmHg	−9.1 ± 5.3	−6.0 ± 4.7	0.003
Major cardiovascular events, *n* (%)	13 (14.1)	21 (24.4)	0.03

With regards to the follow-up outcomes, those patients in the intensive management group were able to demonstrate a lower incidence of major cardiovascular events relative to the standard group (14.1% vs. 24.4%, *p* = 0.03). In particular, the myocardial infarction and heart failure hospitalization rates were lower for patients under intensive management, while stroke and cardiovascular mortality showed a positive trend but were not statistically significant.

### 3.2 Comparative analysis of cardiovascular events

When the cardiovascular outcomes were assessed separately, patients in the intensive management group had fewer myocardial infarctions (5.4% vs. 11.3% %) and were less likely to be hospitalized for heart failure (3.3% vs. 9.0%) than patients in the standard management group. While the stroke rate was lower in the intensive group (4.3% vs. 7.9%), this difference was not statistically significant (*p* = 0.12). Patients in the intensive group also experienced lower cardiovascular mortality (1.1% vs. 3.4%), but the small sample size for this outcome diluted the group difference sufficiently to impair the ability to detect significant differences in this outcome (Table 3).

**Table 3:** Incidence of major cardiovascular events according to achieved systolic blood pressure group.

Outcome	Lower SBP group (*n* = 92)	Higher SBP group (*n* = 88)	*p*-value
Composite major CV events, *n* (%)	13 (14.1)	21 (24.4)	0.03
Myocardial infarction, *n* (%)	5 (5.4)	10 (11.3)	0.11
Stroke, *n* (%)	4 (4.3)	7 (7.9)	0.12
Heart failure hospitalization, *n* (%)	3 (3.3)	8 (9.0)	0.04
Cardiovascular mortality, *n* (%)	1 (1.1)	3 (3.4)	0.27

**Figure 2:**
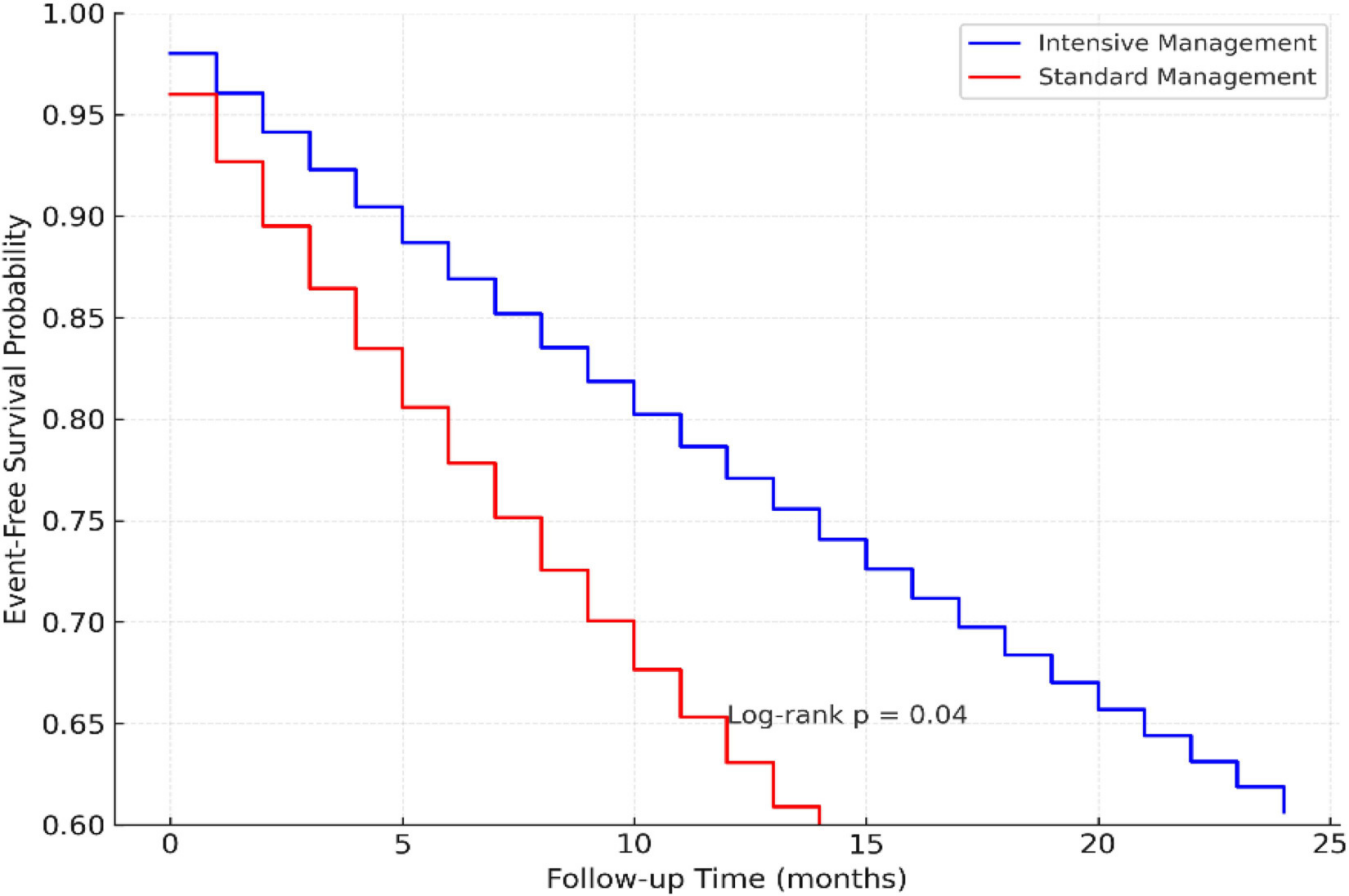
Kaplan–Meier survival curves for primary composite cardiovascular outcomes.

**Figure 3:**
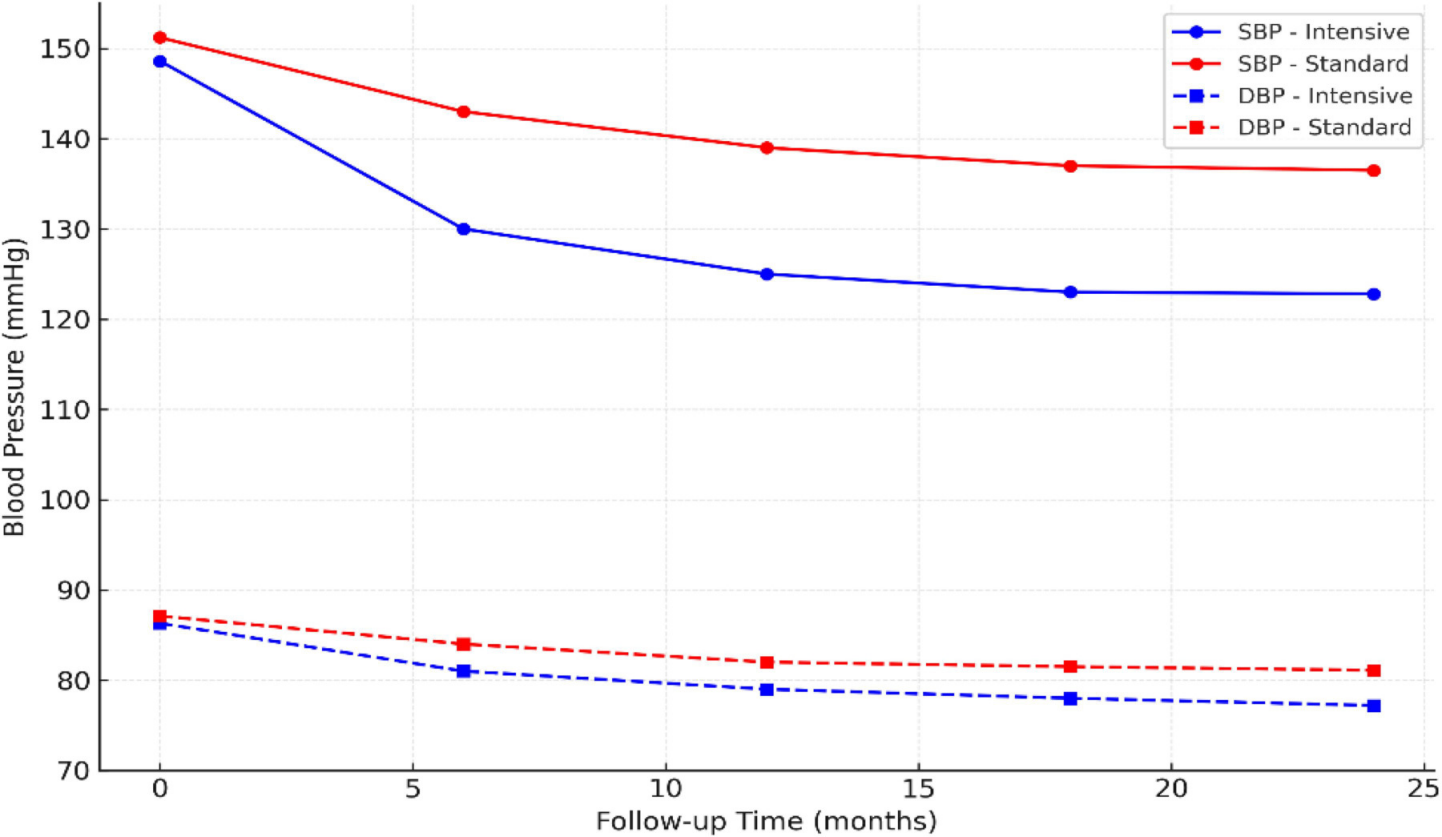
Mean blood pressure trends over time.

Kaplan–Meier survival curves indicated that the intensive management group experienced better event-free survival than the other group during the follow-up period (Figure 2). The log-rank test showed that patients treated with intensive blood pressure targets had significantly lower cumulative incidence of major cardiovascular events compared to patients managed with standard targets (*p* = 0.04) (Figure 3).

### 3.3 Subgroup analysis

Subgroup analyses were conducted to determine whether the benefits of intensive blood pressure management were consistent across the various categories of patients. In the case of patients with diabetes mellitus, intensive management resulted in a significant reduction in composite cardiovascular events (15.2% vs. 28.7%, *p* = 0.02). Likewise, patients with chronic kidney disease in the intensive group demonstrated lower event rates compared to those in standard control (17.5% vs. 30.1%, *p* = 0.04). In patients aged ≥ 70 years, the benefits were less marked, with only a modest trend toward risk reduction, which was not statistically significant (Table 4).

**Table 4:** Subgroup analysis: cardiovascular event rates by achieved systolic blood pressure in specific patient populations.

Subgroup	Lower SBP group (event rate %)	Higher SBP group (event rate %)	Hazard ratio (95% CI)	*p*-value
Diabetes mellitus (*n* = 83)	15.2	28.7	0.58 (0.34–0.96)	0.02
Chronic kidney disease (*n* = 51)	17.5	30.1	0.61 (0.38–0.99)	0.04
Age ≥70 years (*n* = 47)	20.8	27.5	0.75 (0.42–1.34)	0.18
Male sex (*n* = 104)	13.9	23.6	0.64 (0.37–1.09)	0.09
Female sex (*n* = 76)	14.3	25	0.62 (0.33–1.12)	0.08

Interestingly, when patients were grouped by sex, there was no difference in the magnitude of treatment effect, which means men and women gained the same benefits from intensive blood pressure lowering. These data support the hypothesis that patients with metabolic and renal comorbidities stand to gain the most from this form of management, whereas its use in older patients is less defined and requires more scrutiny.

### 3.4 Adverse events

The care of patients with Intensive Management and Standard Management of Blood Pressure Targets was monitored with respect to safety throughout the follow-up period. Adverse events associated with the treatment of the patients in the intensive group were reported as slightly higher than the events reported with the standard group (11.9% vs. 7.2%). The most common side effects were those of treatment, which were classified as hypotensive in nature. 6.5% of patients in the intensive treatment group reported hypotensive episodes as compared to 3.4% in the standard group. Electrolyte imbalance in the form of hyponatremia and hypokalemia was reported as occurring in 3.3% of patients under intensive management of blood pressure. Acute kidney injury was reported in 2.1% of those patients, although in most cases, resolution occurred with dose, and thus, was classified as transient (Table 5).

**Table 5:** Adverse events during follow-Up.

Adverse event	Lower SBP group (*n* = 92)	Higher SBP group (*n* = 88)	*p*-value
Any adverse event, *n* (%)	11 (11.9)	6 (7.2)	0.21
Symptomatic hypotension, *n* (%)	6 (6.5)	3 (3.4)	0.31
Electrolyte imbalance, *n* (%)	3 (3.3)	2 (2.3)	0.68
Acute kidney injury, *n* (%)	2 (2.1)	1 (1.1)	0.57
Therapy discontinuation due to adverse events, *n* (%)	1 (1.1)	1 (1.1)	0.98

The majority of patients in the study were classified as mild, and thus prone to suffer from moderate in severity, which rarely, if at all, resulted in the need for permanent cessation of any form of antihypertensive treatment. The major safety profile suggested there was a positive benefit to risk balance; therefore, intensive blood pressure control for the purposes of ensuring cardiovascular protection was indicated.

To further characterize differences between the groups, we compared the number and classes of antihypertensive medications prescribed during the study period (Table 6). Patients in the lower SBP group received a significantly higher average number of antihypertensive agents (2.9 ± 1.1) compared to those in the higher SBP group (2.2 ± 0.9; *p* = 0.01). Use of ACE inhibitors or angiotensin receptor blockers (ARBs) was also more frequent in the lower SBP group (67.4% vs. 54.5%, *p* = 0.04), as was the use of three or more drug classes (44.6% vs. 21.6%, *p* = 0.003). These findings suggest greater pharmacologic intensity in the lower SBP group, although medication dose and adherence data were not available.

**Table 6:** Antihypertensive medication Use between achieved BP groups.

Medication parameter	Lower BP group <br > (SBP < 120 mmHg, *n* *=* *92*)	Higher BP group <br > (SBP 130–139 mmHg, *n* *=* *88*)	*p*-value
Mean number of antihypertensive agents	2.9 ± 1.1	2.2 ± 0.9	0.01
ACE inhibitor or ARB use (%)	67.4% (62/92)	54.5% (48/88)	0.04
Beta-blocker use (%)	48.9% (45/92)	45.4% (40/88)	0.62
Calcium channel blocker (CCB) use (%)	42.4% (39/92)	37.5% (33/88)	0.49
Diuretic use (%)	37.0% (34/92)	28.4% (25/88)	0.15
≥3 drug classes used (%)	44.6% (41/92)	21.6% (19/88)	0.003

## 4 Discussion

In this retrospective study of 180 high-risk hypertensive patients, we found that those who achieved a lower systolic blood pressure (SBP) level (<120 mmHg) experienced fewer major adverse cardiovascular events (MACE) compared to those with higher achieved SBP levels (130–139 mmHg). Patients in the lower achieved SBP group not only reached lower systolic and diastolic blood pressure targets but also had better event-free survival over a median follow-up of 18 months. The protective role of controlled systolic blood pressure was greatest in individuals with diabetes and chronic kidney disease, and not as strong in patients older than 70 years of age. Although harmful effects, including symptomatic hypotension and mild renal impairment, were more common in the intensive group, they were generally acceptable given the cardiovascular benefits.

Our research suggested that more intense blood pressure lowering was directly correlated with reduced risk of major adverse cardiovascular events (MACE) in patients who had both diabetes and chronic kidney disease, although the effect was diminished among the aged. The findings align with data from randomized clinical and real-world retrospective cohorts (16). in a recent large-scale nationwide cohort study determined that targeting systolic BP <120 mmHg was associated with a lower risk of myocardial infarction, stroke, and heart failure than standard control (adjusted for comorbidities and demographic variables. Similarly (17), in a high-risk Japanese population, demonstrated that with intensive management, composite cardiovascular outcomes were reduced by 25%, thereby further supporting the need for such research to be more widely incorporated into clinical practice. The present findings are in conjunction with these studies, especially with respect to the renal and metabolic subdivisions. A recent study by (18) further strengthened the evidence base, demonstrating that intensive systolic BP lowering consistently reduced cardiovascular events across multiple populations, with relative risk reductions ranging from 20% to 25%. However, elderly patients (>75 years) derived less benefit and were more prone to treatment-related complications, which echoes our findings of attenuated benefit and higher rates of adverse events in this subgroup.

Additionally, a nationwide Chinese cohort by (19) observed that intensive BP lowering not only reduced ischemic stroke and cardiovascular death but also improved overall survival, although the magnitude of benefit varied by comorbidity status. This heterogeneity of treatment effect parallels our observation that patients with diabetes and CKD gained greater cardiovascular protection, highlighting the importance of tailoring treatment targets according to baseline risk (19). Taken together, our study contributes to the growing consensus that intensive blood pressure management is both effective and clinically relevant in high-risk populations, while also reinforcing the cautionary message from recent trials and observational studies that intensive therapy may not provide uniform benefit across all patient groups. In particular, our findings add valuable real-world evidence by demonstrating that, although the risk of adverse events such as hypotension and transient renal impairment is modestly higher with intensive therapy, the overall balance of benefit favors more aggressive treatment in carefully selected patients.

The results of our study also align with the surfacing evidence concerning the impact of intensive blood pressure control on cardiovascular risk. The SPRINT trial showed cardiovascular outcomes for patients who targeted a systolic blood pressure of <120 mmHg (12). They had a lower rate of myocardial infarction, heart failure, and cardiovascular death compared to patients who had a more standard target of <140 mmHg (20). More intensive blood pressure lowering is especially beneficial for people with diabetes and kidney disease. The reduction in cumulative cardiovascular events with aggressive treatment among our patients concurs with these observations and supports the hypothesis that strict goals are more advantageous in high-risk individuals. But, unlike SPRINT, our design was more applicable to real-world settings. We presented patients with a known history of coronary disease, with a combination of blood pressures and starting blood pressures. It's also worth bearing in mind, as we and others have noted, that the benefit of intensive therapy was smaller among older patients (21).

The mechanisms through which more aggressive blood pressure control translates into improved cardiovascular outcomes remain unclear. Lowering systolic blood pressure alleviates the stress of systolic pressure over the arterial walls, softens the walls of atherosclerotic plaque, reduces the aortic pressure against the left heart, and thereby reduces the risk of heart attacks and heart failures. Moreover, it has been demonstrated that aggressive control improves the function of blood vessels, decreases inflammation of blood vessels, and prevents damage to vital organs, especially the kidneys and the brain. Clinically, this aggressive approach to managing blood pressure and tailoring it to the risk factors for each patient pays off. The greater benefit to patients with diabetes and chronic kidney disease argues that this group of patients may benefit the most from more aggressive intervention. The slight benefit to older patients, however, demonstrates how careful the tightrope walk must be between protecting the cardiovascular system and risking a cardiovascular event like low blood pressure and injury to the kidneys. This suggests a need to personalize the treatment response, becoming more aggressive in patients carrying a high cardiovascular burden and more conservative among older individuals, where risk factors are frail.

A major strength of the study we have performed here is, however, its design and its proximity to routine clinical practice rather than a randomized clinical trial environment. This increases the generalizability of our results, since our cohort included patients with many comorbidities (cardiovascular disease, diabetes, and chronic kidney disease), who were usually underrepresented in randomized studies. Although conducted in a real-world setting, this analysis was systematic by electronic medical records and allowed for the increased comprehensive assessment of cardiovascular outcomes and safety events, over a relatively long follow-up (median 18 months). These features correspond to the methodological soundness described in recent large retrospective studies and cohorts. It was one of the studies that furnished pragmatic, real-world evidence around safety and efficacy regarding intensive blood pressure targets in diverse patient populations, illustrating the value of real-world data.

Our study has several important limitations. First, the retrospective nature of the analysis precludes any causal inference. Patients were not randomized to BP targets, and grouping was based on achieved SBP during follow-up. As such, the observed differences in cardiovascular outcomes may be influenced by unmeasured confounding, such as disease severity, medication adherence, or physician prescribing behavior. Second, although we were able to compare the number and classes of antihypertensive medications between groups, we lacked data on drug dose, titration history, and treatment duration, which limits our ability to fully characterize pharmacologic treatment intensity. Third, the sample size was modest, and certain subgroup analyses (e.g., elderly patients) may have been underpowered to detect significant differences. Finally, although the study was conducted in a real-world setting, potential biases related to documentation quality and follow-up completeness must be acknowledged.

Further studies need to be conducted to validate our findings in larger, multicenter prospective cohorts and avoid the bias of a retrospective study. There is a need for trials that are truly randomized controlled and reflect the clinical situation of the real patients, among them primarily geriatric and multimorbid/polypharmacy ones, to find out the best BP targets in different high-risk patient groups. Patients with diabetes and CKD are likely to gain more than other groups; elderly patients may require a less aggressive approach; therefore, trials will need to determine the optimal treatment threshold. Moreover, more attention should be paid to response and adverse effects prediction of treatment based on new biomarkers and imaging tools. Recent studies suggest that some patients with microvascular renal disease may potentially be treated more aggressively based on the presence of vascular stiffness, endothelial dysfunction, and impaired renal microvascular markers. In addition, artificial intelligence decision support programs that are incorporated within an electronic medical record system developed for blood pressure control at the clinical level may help clinicians to more optimally prioritize blood control with the individual risks faced by a given patient, thereby enhancing overall patient safety and efficacy of therapy.

In the future, studies must include robust cardiovascular protection and long-term follow-up to determine the protection's endurance, as well as monitoring adverse effects that arise late. These two gaps must be resolved during future research to determine the most effective ways for improving blood pressure control in patients with the greatest risk, weighing cardiovascular protection and safety vs. benefit imbalance. In the retrospective study on high risk of hypertension, high blood pressure control was significantly associated with a lower major cardiovascular event rate compared to standard care. These benefits worsened with age and were especially notable for those with diabetes and chronic kidney disease. Intensive therapy's adverse event rate was low, and although higher than that of standard care, the events themselves were minor and did not diminish the cardiovascular benefits attained. The research has shown value in restricting blood pressure in more particular groups of people with higher risks of complications. Our findings also acknowledge the concept of nuanced management. This has particular relevance to geriatric patients, where overzealous management, in an attempt to lower blood pressure, could yield unintended consequences. Overall, this research speaks to the importance of focused blood pressure management, while it also highlights the need for future research to tighten the boundaries for more meshed, patient-centered approaches.

## 5 Conclusion

In this retrospective cohort of high-risk hypertensive patients, lower achieved systolic blood pressure levels were associated with a significantly reduced risk of major adverse cardiovascular events. These associations were particularly notable in patients with diabetes and chronic kidney disease, suggesting that these populations may benefit most from tighter blood pressure control. However, given the non-randomized design of the study and the lack of detailed treatment intensity data, these findings should be interpreted as hypothesis-generating rather than definitive evidence of treatment effect. Further prospective studies are warranted to clarify optimal blood pressure targets in different high-risk patient groups and to evaluate the long-term safety and efficacy of more intensive blood pressure control strategies in real-world settings.

## Data Availability

The raw data supporting the conclusions of this article will be made available by the authors, without undue reservation.
